# Supplementary material for: Identification of photoperiod-induced specific miRNAs in the adrenal glands of Sunite sheep (Ovis aries)
Source: Front Vet Sci. 2022 Jul 22;9:888207. doi: 10.3389/fvets.2022.888207 (PMC9354845; doi:10.3389/fvets.2022.888207)
Supplement: Supplementary file 1 [file Data_Sheet_1.ZIP › Supplementary file/miRDeep2 file.zip!/miRDeep2 file/Know_Structure/SPLP42A1/oar-mir-1185.pdf]

miRBase precursor : oar-mir-1185
Total read count : 160
oar-miR-1185-5p read count : 60
oar-miR-1185-3p read count : 99
remaining reads : 1

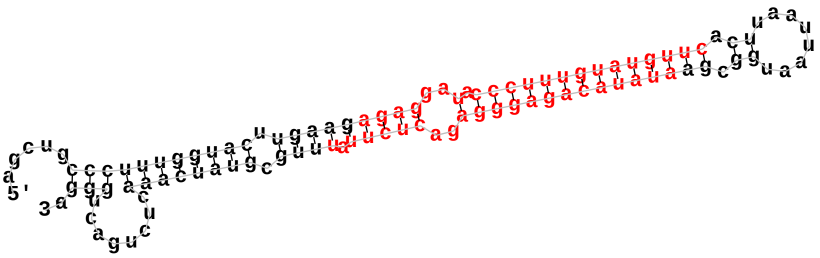

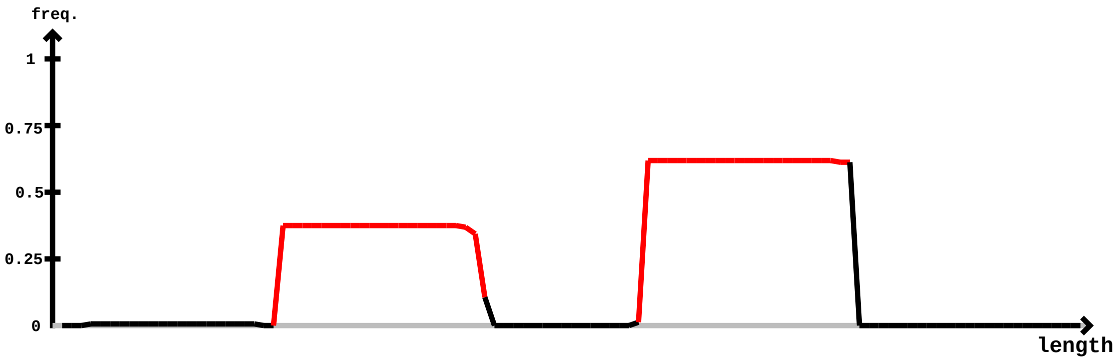

oar-miR-1185-5p

oar-miR-1185-3p

5'- agcugcccuuugguacuugaagagaggauacccuuuguauguucacuuaauuaauggcgaauauacagagggagacucuuauuugcguaucaaacucugacuggga -3'	exp		
.....((((((((((.(((((((((..(.(((((((((((((((.((........)).)))))))))))))))..))))).)))).))))))))........))).	reads	mm	sample
..cugcccuuugguacuuga..	1	0	seq
......................agaggauacccuuuguaug...	1	0	seq
......................agaggauacccuuuguaugu..	4	0	seq
......................agaggauacccuuuguauguu...	38	0	seq
......................agaggauacccuuuguauguuc..	17	0	seq
....aauauacagagggagacucuuau........................	2	0	seq
....auauacagagggagacucuu..........................	1	0	seq
....auauacagagggagacucuuau........................	96	0	seq
